# Role of Alternative Polyadenylation during Adipogenic Differentiation: An *In Silico* Approach

**DOI:** 10.1371/journal.pone.0075578

**Published:** 2013-10-15

**Authors:** Lucía Spangenberg, Alejandro Correa, Bruno Dallagiovanna, Hugo Naya

**Affiliations:** 1 Bioinformatics Unit, Institut Pasteur Montevideo, Montevideo, Uruguay; 2 Instituto Carlos Chagas, Fiocruz-Paraná, Curitiba, Paraná, Brazil; 3 Departamento de Producción Animal y Pasturas, Facultad de Agronomía, Universidad de la República; Harbin Institute of Technology, China

## Abstract

Post-transcriptional regulation of stem cell differentiation is far from being completely understood. Changes in protein levels are not fully correlated with corresponding changes in mRNAs; the observed differences might be partially explained by post-transcriptional regulation mechanisms, such as alternative polyadenylation. This would involve changes in protein binding, transcript usage, miRNAs and other non-coding RNAs. In the present work we analyzed the distribution of alternative transcripts during adipogenic differentiation and the potential role of miRNAs in post-transcriptional regulation. Our *in silico* analysis suggests a modest, consistent, bias in 3′UTR lengths during differentiation enabling a fine-tuned transcript regulation via small non-coding RNAs. Including these effects in the analyses partially accounts for the observed discrepancies in relative abundance of protein and mRNA.

## Introduction

Mesenchymal stem cells (MSCs) are able to differentiate to mutiple cell types including those in bone, ligament, muscle and connective tissue [1] among others and are thus the focus of stem cell-based therapies. Tissue engineering [2], therapy for degenerative and autoimmune diseases [3], [4] and cardiac tissue repair [5], [6] are some of the areas of focus in adult stem cell research. Although much progress has been made, the regulatory processes controlling MSC differentiation remains poorly understood. Adipose derived human MSCs are easily isolated from pools of cells resident in vascular stroma of adipose tissue. Since adipose tissue is ubiquitous and easily accessible with minimally invasive procedures [7], it is an ideal resource for research and development of cell-based therapy. Understanding MSC commitment to differentiation to a specific cell type is essential for the successfully repair or regeneration of injured tissues. The switch from self-renewal to differentiation is regulated by many factors including cytokines, growth factors and extracellular matrix components present in a given microenvironment [8]. Nevertheless, the transcriptional and post-transcriptional regulatory processes remain not fully understood.

Gene expression analysis has provided great insights into the regulatory networks determining self-renewal and differentiation processes [9], [10]. Deep sequencing techniques have also played a key role in clarifying the complex mechanisms involved. Regulation is at both the transcriptional [11] and post-transcriptional [12], [13] levels. Also non-coding elements are involved [14] in the regulatory machinery [15]. In order to address post-transcriptional regulation, many groups are focusing on sequencing mRNAs associated to translating polysomes and comparing them with total RNA [12], [13], [16].

Expression analysis with deep sequencing methods enables the distinction of alternative transcripts of the same gene. In this context, the focus is shifted from analyzing genes as an entity (represented by a single canonical transcript) towards an alternative transcript usage model, non-coding RNAs (e.g., miRNAs), alternative splicing, 3′UTR switching, polyadenylation [17], [18], etc. Alternative polyadenylation (APA) results in subpopulations of transcripts differing in 3′UTR length, which makes them more or less susceptible to the regulation by miRNAs (shorter 3′UTR might have fewer miRNA binding sites) [19], [20]. A recent study has shown a role for APA in muscle stem cell development. The Pax3 protein represses differentiation in that transcripts can be targeted by mir-206. Boutet *et al.* [18] showed that different muscle tissues process Pax3 transcripts differently through APA, in which transcripts were differentially targeted by miR-206 based on 3′UTR length. In turn, different Pax3 protein levels result in functional changes in muscle stem cell behavior. Other groups assessed this type of mechanism in a global way, analyzing 3′UTRs length patterns of all genes in different scenarios. Sandberg *et al.* showed a global shortening of 3′UTRs in proliferating murine CD4+ T lymphocytes [21], and Kolle *et al* showed human embryonic stem cells to have extended 3′UTRs. The latter study also found alternative gene model usage [13]. In addition, Ji and collaborators reported that mouse genes tend to express longer 3′UTRs during the progression of embryonic development [22].

In the present work, we focus on post-transcriptional regulation during adipogenesis, specifically analyzing transcript usage differences based on 3′UTR length. We analyze data previously obtained using RNAseq [12] to study the initial phases of adipocyte differentiation of adipose-derived human mesenchymal stem cells (hASCs). Total mRNA (total) and mRNAs associated with translating ribosomes (polysomal fraction) were sequenced at two time points: 0 and 3 days after induction. We found that 3′UTRs tended to be longer after cells were induced, thereby potentially providing more miRNA binding sites. A mean difference of 18 bases in transcript length was found in induced versus control conditions. In our previous study, based on a subset of the proteomic data of Molina *et al.* [23], we found a low correlation between protein and corresponding mRNA changes. Standard linear models predicting changes in protein levels based only on mRNA changes were inaccurate. Here, we propose linear models that incorporate the effect of miRNAs on protein changes, which substantially improve the correlation between protein and mRNA change. Furthermore, our linear models indicate several miRNAs that could potentially be involved in post-transcriptional regulation of genes relevant for adipogenesis. Moreover, we also observed that genes previously described as involved in the differentiation process (Plurinet genes [24]) are enriched in longer 3′UTR in the induced condition.

## Results

### 1 Global analysis of differential transcript usage

Previous studies have shown that the use of alternative polyadenylation sites, which generates transcripts with varying 3′UTR length (shorter or longer), are associated with cells having higher proliferation rates [21], [22] (those generally having shorter 3′UTR), with cells undergoing differentiation [13] (longer 3′UTR) and with post-transcriptional regulation events in general. We determined alternative transcript usage by comparing the proportions of FPKM of each transcript for IN (induced samples, differentiating cells) vs. CT (control samples, undifferentiated cells). Analysis was done with total and polysomal fractions (see 2), however, total RNA was analyzed in greater detail to more accurately recover all alternative transcripts. Transcripts destabilized by miRNA are not expected to be associated with polysomes.

A preliminary global analysis of our data showed that the average 3′UTR length, weighted by the proportion of transcripts used for each gene, differed under IN compared with CT conditions. The mean difference was 18 bases, and 11 bases when outliers were excluded. In this context, we defined outliers as 3′UTRs with an average difference between conditions (IN–CT) longer than 1 kb. We excluded extreme values to avoid a bias in the determination of the mean (only for these calculations). Both lengths (18 and 11) are sufficient for generation of an additional miRNA binding site (see Discussion). Extension of 3′UTR regions was found in 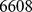 genes (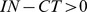, weighted by the proportion of transcripts), whereas 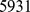 had shorter 3′UTRs (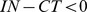). As such, we observed a tendency for longer 3′UTR under IN conditions compared with CT (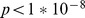, Wilcoxon test). We tested our data using the Cochran-Mantel-Haenszel (CMH) statistic, as in Fu *et al.* [25] to assess the significant of the differences observed. Since several genes have more than two transcripts and the length of the 3′UTR is a quantitative variable, the linear trend alternative to independence test [26] is more accurate than a standard 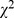 test. CMH determines a trend value for each gene, based on a Pearson correlation, with a corresponding p-value. In our setup, a positive correlation is observed if there is a tendency for longer 3′UTRs under IN conditions and a negative correlation for longer 3′UTR in CT. From the 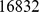 genes tested, 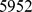 displayed a negative trend, 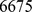 a positive trend and 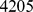 showed no trend. Tendencies are based on the calculated correlation values needed for the CMH test. Furthermore, 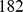 genes were significant at an FDR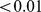. Of the significant 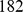 genes, 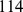 had a positive correlation value and 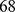 a negative one. This difference is again significant (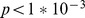, Wilcoxon test). In summary, we found that there is a modest but consistent tendency to use alternative transcripts with longer 3′UTR under IN conditions compared with CT in our dataset.

Trends observed in polysomal fractions were similar to those in total RNA fractions, however, the number of genes were smaller: 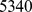 genes had a negative trend (length 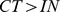), 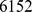, a positive trend (length IN>CT) and 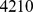 no trend (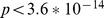). These trend results are also based on the correlation values used for the CMH test. Differences in the distribution of gene trends for total and polysomal fractions were significant (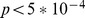), but were relatively small considering the large numbers compared. Of 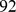 significant genes at FDR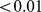, 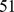 had positive correlation values and 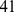 negative values. A number of significant genes, each having at least 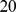 nucleotides of 3′UTR length difference between conditions, were found in both total and polysomal fractions (positives and negatives). The overlap list of negative genes includes: ARL6IP5, COL1A2, RPL23, CD59, THBS1, TMED9, SPARC and MFAP5, and the positive list includes: DCN, BRK1, OSTC, PEBP1, BNIP3L, SAR1A and LSM6.

The observed mean difference in whole transcript lengths between conditions was 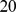 bases, considering all 3′UTRs, and 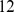 bases without outliers (defined as before). Interestingly, the correlation between trend statistic for total and polysomal fractions was very low, 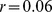 (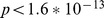), pointing towards important differences in post-transcriptional regulation.

### 2 Large fold change differences between mRNA and proteins

Large differences can be observed between mRNA and protein products in eukaryotic cells. This is due to various types of post-transcriptional regulation including tRNA and ribosome availability, regulation by small non-conding RNAs and transcripts nucleotide composition. However, in general a reasonably good agreement (in logarithmic base) is expected [27], [28]. We previously correlated protein fold changes (in mouse) determined by SILAC (Molina *et al.* [23]) and our human RNAseq data [12]. We found a relatively high correlation between our RNAseq data and a subset of Molinas data, consisting of a group of secreted proteins. However, we were unable to find a high correlation with the entire dataset, which also included nuclear proteins. Using the same data set, we addressed the reasons behind the low correlations observed between mRNA and protein fold changes. In brief, our RNAseq dataset consists of two sets: RNAseq of total RNA (total) and of polysome associated RNA (polysomal). The samples were hADS cells taken at time point 0 (control; CT) and three days after adipogenesis induction (induced; IN). Molina *et al.* measured 3T3-L1 murine stem cell protein levels at different time points during adipogenesis: day 0, 1, 3, 5 and 7. Ideally, such comparisons would be more appropriate comparing experiments from the same species, however, Molina's dataset was the most suitable available for comparison with our RNAseq analysis (see Materials and Methods). To the best of our knowledge, studies on adipogenesis comparing different species have not been reported. However, embryonic stem cell pluripotency is established and maintained by a largely conserved regulatory network in eutherian mammals [29]. Other studies have shown conserved genes and pathways involved in mammary gland development in human and mouse similarly governing cell-fate decisions and differentiation processes [30].

**Figure 1:**
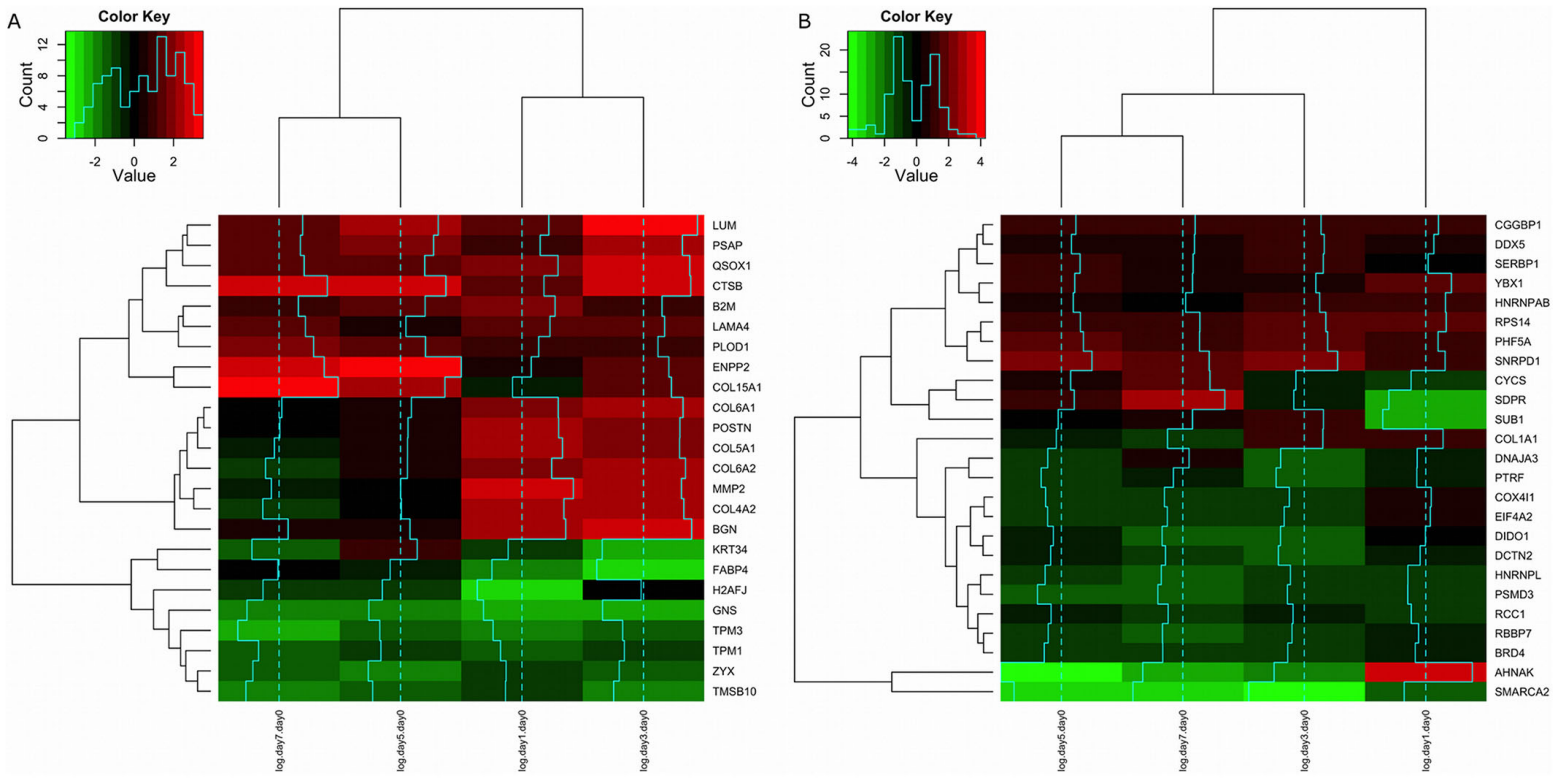
Heatmap of the residuals of the model logFC 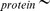
logFC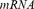. Protein levels (logFC) of the set of secreted proteins are compared against the logFC of our data set and the residuals of the linear model analyzed; polysomal fraction (A) and total fraction (B). All time points are considered: day 1, 3, 5 and 7 (dendrogram on the top). Genes are on the rows (dendrogram on the left). Only data for genes with large absolute residuals are shown.

A linear model for logFC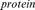 values (log fold change of protein, e.g. 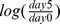) versus our logFC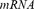 values (log fold change values of mRNA, 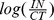) was fit for each time point in the experiment of Molina *et al.*, and residuals analyzed. Such differences (residuals of the corresponding linear model) were very large for several genes. Fig. 1 shows the differences in logFC for each time point (day 1, 3, 5 and 7) in the secretome dataset (nuclear in Fig. S1). Only those genes with the greatest differences are shown, and both RNA fractions are considered (A polysomal and B total). Genes clustered into two groups: negative differences (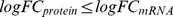) are shown at the bottom of Fig. 1 (green) and positive differences above (red). The large differences suggest post-transcriptional regulation of several genes potentially by small non-coding RNAs, especially miRNAs. Linear models were constructed taking into account alternative transcript usage between conditions and characterizing miRNA binding sites involved. We discuss these results in the next subsections.

### 3 Alternative transcripts and miRNAs help explain protein fold changes

We analyzed the effect of miRNAs targeting 3′UTR of alternative transcripts in the fold change of proteins by linear models. The base model included only logFC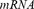 (and the intercept) as predictor variable for the logFC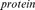. miRNA target sites were then included in order to increase the variance explained by the model. Of the 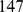 secreted proteins analyzed by Molina *et al.*, 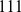 genes were represented in our expression dataset (total and polysomal RNA), and of the 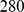 nuclear proteins, 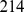 were found in our set. In addition, we determined the relative transcript abundance per gene in our dataset (using *cuffdiff*, see 5). Once we established the miRNAs targeting those transcripts (weighting by transcript usage) and the logFC values for each gene, we predicted the effect of each miRNA on protein level. Hereinafter, when we mention models “including/considering miRNAs”, we are referring to models, which incorporate the effect of the differences in miRNA target sites. First, models including each miRNA individually were constructed (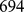 miRNAs in total), then all combinations of two to five miRNAs were included in models. The best models were selected based on BIC (Bayesian Information Criterion).

Table 1 shows linear model results for secreted and nuclear proteins with polysomal and total RNA fractions. The base model (the effect of logFC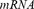 on protein change without considering any miRNAs) is shown, as well as two single miRNA models (per comparison: polysomal/total and secreted/nuclear) and the best model by BIC (including one or two miRNAs).

**Table 1:** Linear model results for secreted and nuclear proteins at day 5.

SECRETOME				NUCLEAR			
Polysomal RNA				Polysomal RNA			
logFC	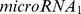	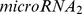	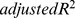	logFC	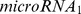	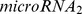	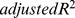
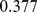 †	-	-	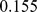	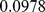	-	-	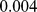
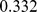 †	miR-130a†	-	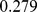	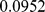	miR-185*†	-	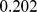
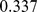 †	miR-130b†	-	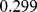	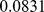	miR-20b*†	-	0.175
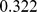 †	miR-130b†	miR-558†	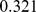	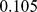	miR-16-2*†	miR-185*†	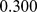

Results for applying linear models to the data at day 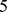 secreted and nuclear proteins. Both RNA fractions are considered. For each subtable (e.g. secretome-polysomal) the first row shows the results for a linear model without considering microRNA effect (the standard model: 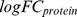 vs. 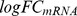). The 2*^nd^* and 3*^rd^* row represent the values for univariate models, including the effect of only one miRNA. We selected the two most significant miRNAs. The last row shows the (multivariate) best model as determined by the BIC value. In several cases the best model is not multivariate, especially since BIC penalizes the number of parameters.

† means a significance level of 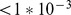.

**Figure 2:**
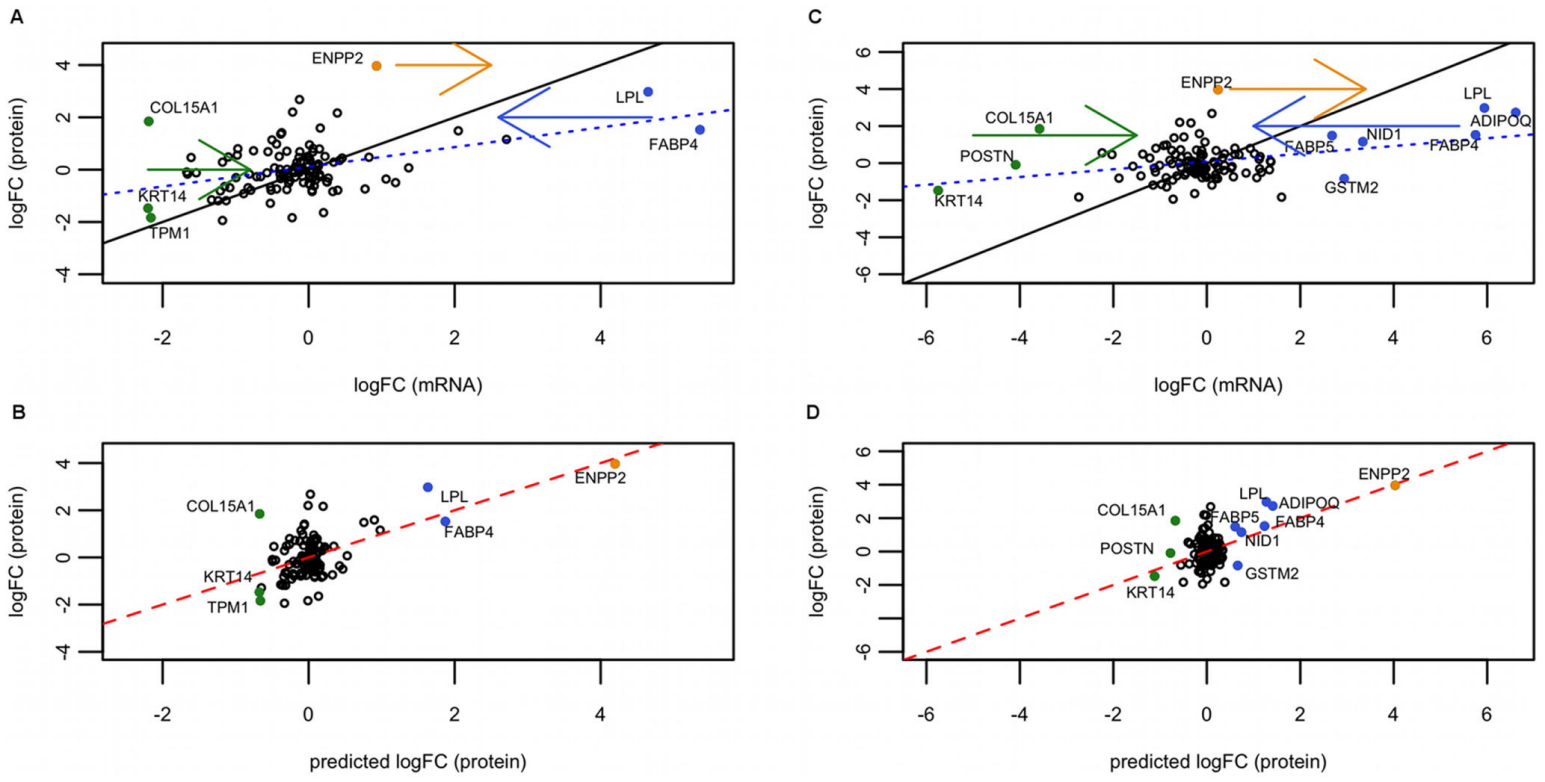
Bootstrap to asses our results for each RNA fraction and each protein set. Bootstrap results for total RNA fractions are shown in A (nuclear) and B (secretome). Polysomal fraction is shown in C (nuclear) and D (secretome). For each such pair of conditions, we performed a bootstrap analysis as explained in 0.6. For each miRNA we permute the values of the genes and calculate the explained variance from the resulting linear model. This procedure is repeated 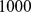 times. The y-axis represents how many times the “true” miRNA wins over the random model. The x-axis represents all miRNAs. The colors, from red to green, represent the explained variance from the current “true” model. It can be observed that the miRNAs win almost all times (the larger bars, almost reaching 1), explain the larger variance, and hence produce the best models (red).

The variance explained by the models increases substantially when the effect of specific miRNAs is incorporated. For example, for polysomal secreted proteins, the base model explains 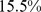 of the variance, while 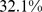 is explained by the two-miRNA model. The effect of miR-130b and miR-558 on the logFC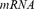 more accurately reflects the observed protein logFC. These miRNAs may have an important regulatory role in adipogenesis. Similar results were obtained with the remaining datasets. In addition, we also found that variances explained by polysomal fraction models (secretome and nuclear) were in general higher than those using total RNA (Table 1). This can be explained by the reduced effect on mRNA destabilization in polysomal mRNAs (they are already associated with polysomes). Finally, Table 2 shows all miRNAs that were significant at an FDR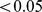 in single miRNA models at day 5, in the different datasets. Several of these miRNAs (underlined in the table) were previously found to be involved in adipogenesis [31]. To assess the possibility that our results were due to random sampling on the miRNA matrix, we performed a bootstrap analysis as described in section 6. Our results ruled out this possibility, since for all significant miRNAs, much less than 5% of random models had explained variances comparable to BIC-selected “true” models. Fig. 2 shows an analysis indicating how many times each miRNA wins, comparing explained variances using “true” over random models (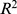 values are color coded). The miRNAs that win at least 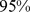 of the times generate the best fitting models (more variance explained), and are shown in red. These miRNAs (red) could be distinguished from those winning in random models (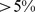). Explained variances for both miRNA groups were compared and the differences were found to be statistically significant (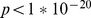, Kruskal-Wallis test); miRNAs winning in true models (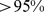 of the times) usually explain much more variance than miRNAs winning in random models (see Fig. S2).

**Table 2:** Significant miRNAs at day 5 as obtained from the linear univariate model.

	Polysomal RNA	Total RNA
secreted	miR-103,miR-107,miR-130a,miR-130b	miR-103,miR-107,miR-130a
	miR-142-3p,miR-144,miR-148a	miR-130b,miR-142-3p,miR-144
	miR-148b,miR-150*,miR-152,miR-15a	miR-150*,miR-152,miR-15a
	miR-15b,miR-16,miR-190b,miR-195	miR-190b,miR-19a,miR-19b
	miR-19a,miR-220c,miR-28-3p,miR-29a	miR-210,miR-220c,miR-26a
	miR-29b,miR-29b-2*,miR-29c,miR-301a	miR-26b,miR-27a*,miR-28-3p
	miR-301b,miR-302a,miR-302d,miR-338-5p	miR-29a,miR-29b,miR-29b-2*
	miR-33a,miR-33a*,miR-33b,miR-340	miR-29c,miR-301a,miR-301b
	miR-486-5p,miR-509-5p,miR-510,miR-551b*	miR-338-5p,miR-33a,miR-33a*
	miR-553,miR-558,miR-569,miR-574-5p	miR-340,miR-361-5p
	miR-589*,miR-628-5p,miR-633,miR-672	miR-486-5p,miR-509-5p
	miR-768-3p,miR-768-5p,miR-891b	miR-510,miR-551b*,miR-553
		miR-558,miR-569,miR-574-5p
		miR-575,miR-582-3p,miR-587
		miR-589*,miR-604,miR-607
		miR-628-5p,miR-672
		miR-768-3p,miR-768-5p,miR-891b
nuclear	miR-143*,miR-16-2*,miR-185*,miR-20b*	miR-100,miR-106b,miR-10b*,miR-185*
	miR-346,miR-372,miR-378*,miR-587	miR-193a-5p,miR-222*,miR-28-5p
		miR-372,miR-433,miR-507
		miR-523,miR-548b-3p,miR-551b
		miR-576-5p,miR-621,miR-885-5p

Set of significant miRNAs in each data set. Underlined miRNAs correspond to those found in Zhang *et al.* (revision on miRNAs involved in adipogenesis) [31].

### 4 Consequences of including miRNAs and alternative transcripts

**Figure 3:**
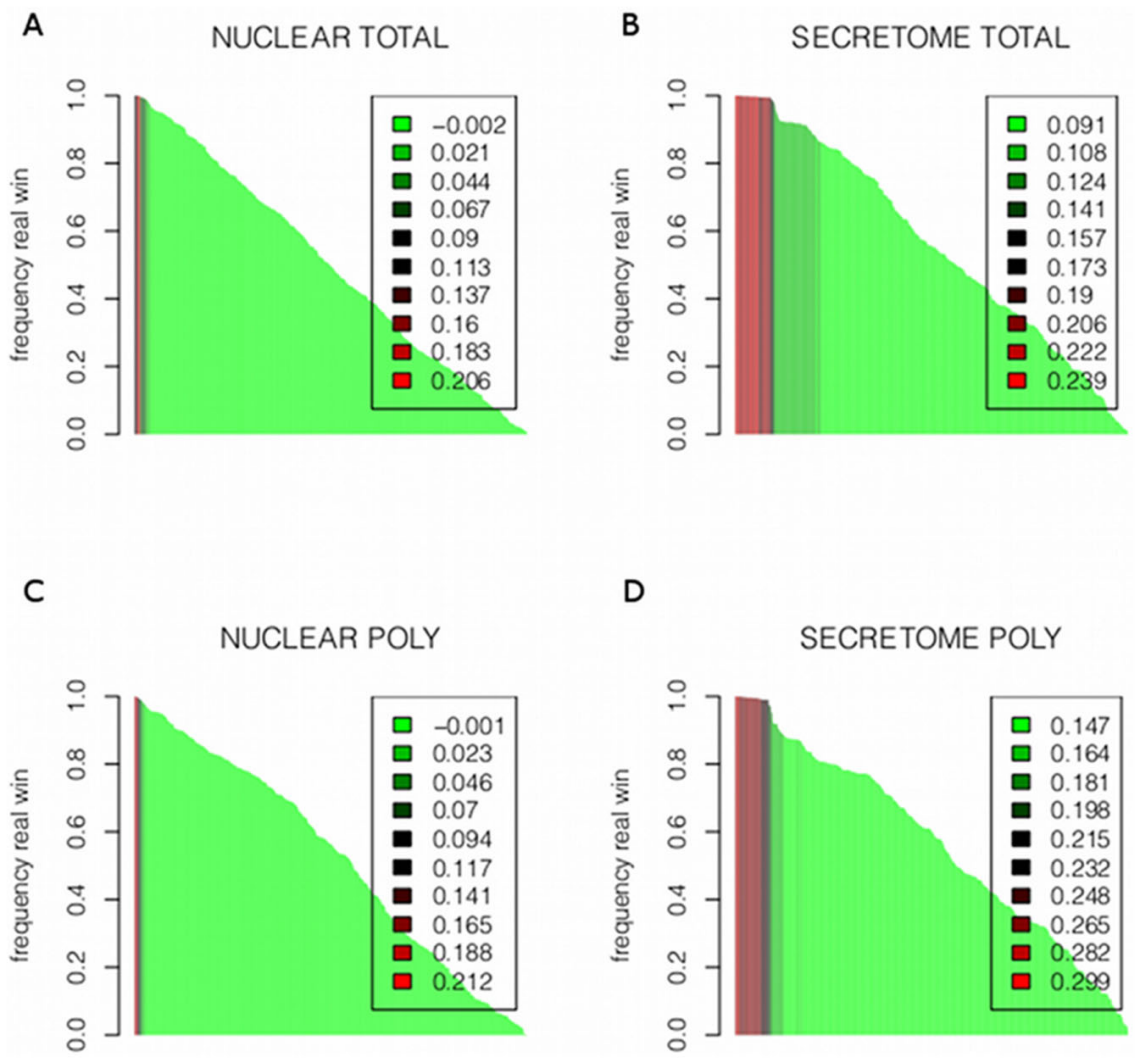
Linear models for day 5 secreted proteins represented graphically. (A, B) Polysomal fraction, (C, D) total RNA. (A) and (C): plot representing logFC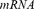 against logFC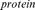. The dashed blue line is the best fitting line of the base model, 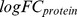 against 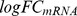. The straight black line is the identity line (so you get an idea of the real coefficient of the model). The colored full dots are genes, which are moved after applying the model with miRNAs. Hence, they represent genes that are better explained by our model. The arrows indicate the direction of the movement. (B) and (D): plot representing our linear model including miRNA effect. In this case, the best (multivariate) model is shown: miR-130b and miR-558 (polysomal) and miR-150* (total). Full dots are the genes that were corrected by our model, being now closer to the protein prediction line of the model (red full line). Black identity line concurs with the red line. Note that the abscissas of (A) and (C) seem to have a compression of range with respect to the plots below, (B) and (D). This is not a compression, since they are different x-axis: (A) and (C) hold logFC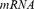 values, while (B) and (D) logFC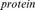.

While the effect of logFC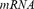 is significant for the secretome set (both fractions), it is not for the nuclear set (both RNA fractions), as shown in Table 1. Significant logFC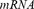 coefficients are higher for polysomal than for total RNA, which is expected since polysomal RNA reflects protein levels more accurately. Fig. 3 summarizes results for the best BIC models for the log-fold change in secreted proteins on day 5 with respect to day 0, for polysomal and total RNA. Fig. 3, (A) and (C) show the distribution of genes when comparing logFC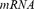 with logFC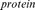 not including the miRNA effect (base model). Fig. 3, (B) and (D) show the model including the effect of miR130b and miR-558 (polysomal) and miR-150* (total). While the base model performs poorly in predicting behavior of several genes (colored dots), in that they deviate from the predicted model line, our model shifts them towards a more expected position. In addition, among the shifted genes several established adipogenesis genes were found: FABP4, FABP5, LPL and ADIPOQ.

The coefficient for logFC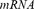 is low in the base model for both RNA fractions, ca. 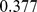 (polysomal) and 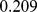 (total). This coefficient decreases even more in our models. This indicates a range compression comparing protein fold-change with mRNA fold-changes (in log-log scale). This might be unexpected, however, translational efficiency (the number of protein molecules produced per mRNA molecule) may decay with the number of transcripts (see Appendix S1 (B) for more details). In fact, several studies have shown a decrease in translational efficiency [27], [28], [32], observed as a linear trend in the dot plot of absolute protein quantity vs mRNA quantity. Furthermore, as we show in Appendix S1 (A), the slope of this relation (1 indicating no decreasing translational efficiency with mRNA quantity, and 0 a complete decrease) is identical to the coefficient of logFC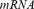 in the linear models we have fit here.

Regulatory features we found help to explain protein level changes seen during adipogenesis, even though we used a limited data set. For this reason, in addition to analyzing significant miRNAs acting as predictor variables in protein-mRNA logFC relationships, we also analyzed the distribution of all miRNAs in all genes (with RNAseq data) having alternative transcripts.

### 5 Multiple miRNA functioning together in regulation

Evidence shows that multiple miRNAs may act together to co-regulate specific genes for normal function [33]–[36]. We investigated co-occurrence of miRNAs in our data set, and found established as well as novel regulatory correlations between them. In addition to the co-occurrence in the linear models described before, we now explored the correlation of miRNA occurrences in the different transcripts analyzing the presence/absence matrix of miRNAs by transcript, weighted by transcript usage differences between IN and CT inside genes (see subsection 7). Based on the total RNA fraction (reflects status of all transcripts, e.g. before degradation) we observed some miRNA pairs with significant correlations. We describe four of the cases found in our study. In all cases, we restricted our analysis to transcripts (rows in the matrix) in which at least one miRNA (of the two per comparison) is present and we compared the correlations obtained with the presence/absence matrix (1 and 0) with the values obtained with the matrix weighted by transcripts usage. First, the presence/absence matrix for miR-204 and miR-211 target sites was considered and a correlation was determined 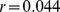 (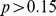). When using the weighted matrix, we obtained a correlation value of 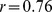 (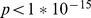). Similarly, for transcripts targeted by miR-17 and miR-93, the correlation using the presence/absence matrix was 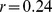 (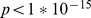), whereas the correlation with the weighted matrix was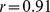 (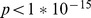). For transcripts targeted by miR-17 and miR-20a a negative correlation is observed using the presence/absence matrix (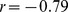, p-value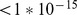), however considering weighted data a significant positive correlation is observed (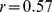, 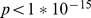). Pair miR-34 and miR-449 presents a negative correlation in both cases (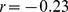, 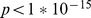 and 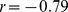 presence/absence matrix, 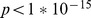 for our weighted data).

### 6 Alternative transcripts in relevant genes from other sources: PluriNet genes

The PluriNet is a protein-protein network with 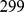 members common to pluripotent stem cells based on gene expression profiles of 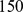 human cell samples. Such molecular network is believed to be involved in the differentiation and self-renewal of pluripotent stem cells [24].

**Figure 4:**
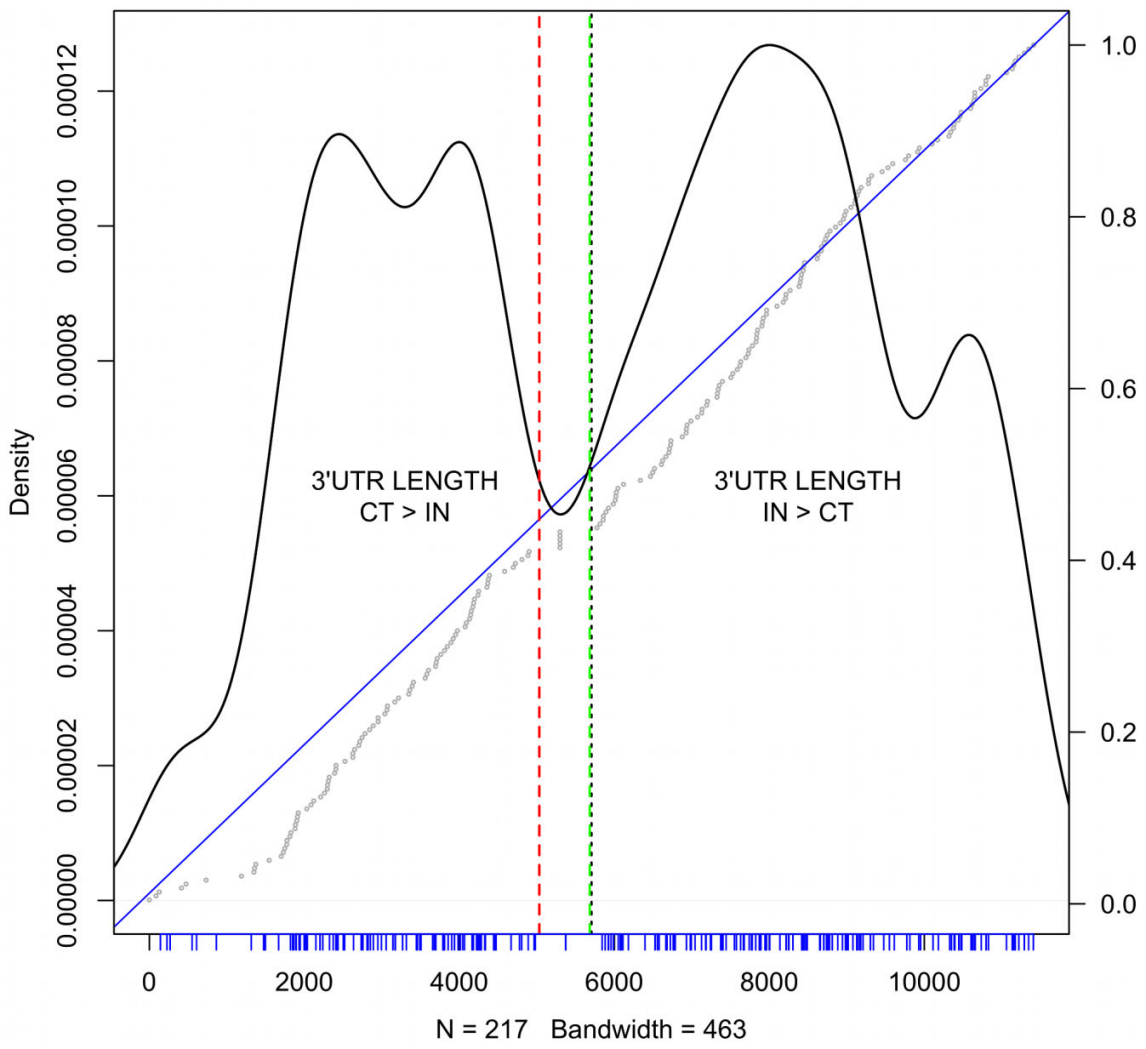
3′UTR differences for PluriNet genes. On the x-axis one observes the ranking of 3′UTR lengths as determined in section 1 of all genes used for logFC calculations in the total RNA fraction. The ranking of genes belonging to the PluriNet are shown as densities (y-axis on the left). Negative lengths (CT>IN) lie to the left of the red dashed line. Positive values are to the right of the green dashed line. The wide space between those lines correspond to genes with no differences in 3′UTR length. The median of the rankings is represented as a doted black line. Tick marks in blue represent the ranking positions of the PluriNet genes. On top of the density plot the cumulative distribution of rankings is shown. The straight blue line has slope 1 and intersect 0. Gray dots represent the cumulative ranking of the PluriNet genes. The y-axis to the right indicates the meassure of this cumulative ranking. An under-representation of PluriNet genes with high negative values and a slight over-representation of positive values is observed. Moreover, only marginal PluriNet genes are presenting values of 0.

We investigated 3′UTR length distribution of PluriNet transcripts for IN vs CT conditions. Similar trends were observed for the total and polysomal fraction. We found that positive differences correspond to longer 3′UTR under IN conditions, and negatives the converse situation (zero indicates no differences), when considering the weighted differences in length (as determined in 4). We first ranked all genes by 3′UTR length differences, and identified PluriNet genes within the ranking. As shown in Fig. 4, PluriNet genes accumulated near small negative differences but distributed evenly for all positive values. Of the 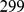 Plurinet genes, 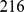 were found in our dataset. 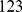 had positive differences in length (3′UTR longer in IN) and 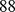 negative (3′UTR longer in CT) with 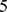 having no differences. GO analysis of the 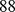 negative genes resulted in the following over-represented terms: metabolism of non-coding RNA (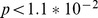), snRNP assembly (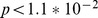), loading and methylation of Sm proteins onto SMN complexes (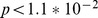), RC complex during G2/M-phase of cell cycle (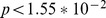). In the set of positive correlated genes, one enriched term was found: nuclear part (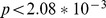).

Interestingly, according to the Cochran-Mantel-Haenszel statistic (with FDR<0.01) the following PluriNet genes showed significant 3′UTR length differences between IN vs CT: PSMA3, PSMA4, PSME3, proteasome assembly (subunits and activator), HSPA8 (heat shock 70 kDa protein 8), SNRPF (small nuclear ribonucleoprotein polypeptide F), SUMO1 (small ubiquitin-like modifier which promotes SUMOylation), TMEM258 (transmembrane protein 258) and SNRPE (small nuclear ribonucleoprotein polypeptide E). Only SNRPE had a positive correlation, while the others had a negative correlation.

## Discussion

**Figure 5:**
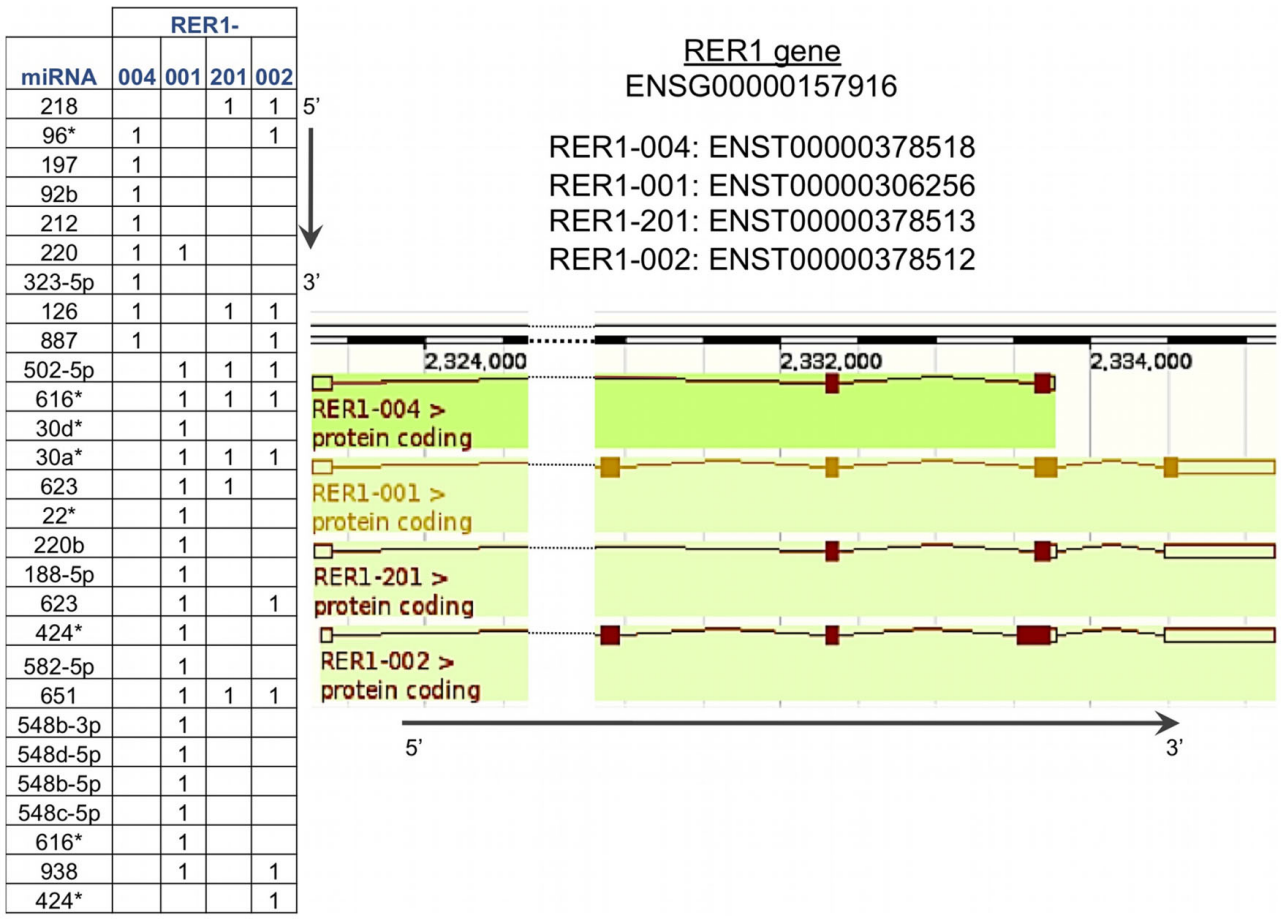
An example of how different microRNAs binding sites arise from alternative transcripts. The table shows the presence of the miRNAs in the transcripts. The longer the 3′UTR the more binding sites are seen.

We previously showed important differences in mRNAs changes comparing polysomal and total fractions during adipogenesis [12]. Furthermore, mRNA changes were poorly correlated with observed protein changes during differentiation [23]. Altogether, these results point to a very important role for post-transcriptional regulation in adipogenesis. To gain deeper insight into the mechanisms involved, we explored the differences observed in alternative transcript usage focusing on differences in the 3′UTR regions. These are relevant since they have well-known regulatory features, particularly involving small non-coding RNAs. An example showing how different miRNA binding sites can be generated in the 3′UTR of alternative transcripts is shown in Fig. 5. The gene illustrated is RER1, which is one of the significant genes in the polysome fraction in this study having alternative transcripts during adipogenesis. As indicated longer 3′UTRs may have additional miRNA binding sites.

Our results show that significant differences in transcript isoforms arise by APA during adipogenesis. A trend towards longer 3′UTR was observed in both RNA fractions, total (18/11 bases) and polysomal (20/12 bases). We proposed that this small differences in length were still sufficient for the generation of new miRNA binding sites. We tested this, by analyzing the pairwise differences between the 3′UTR length of transcripts and the corresponding differences in miRNA binding sites, for each gene. Our preliminary analysis showed that for the differences of interest (20, 18, 12 and 11 bases), out of the 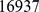 genes analyzed, 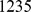, 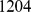, 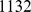 and 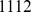 genes, respectively, differed in at least one miRNAs binding site.

The difference in the total RNA fraction is also consistent with the number of genes displaying a positive trend (3′UTR length IN>CT), which is significantly higher than those showing a negative trend. Regarding trend-length differences comparing IN and CT conditions, 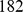 genes showed statistically significant trends (FDR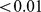): 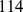 had a positive correlation value and 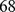 a negative value. Very similar trends were also observed for correlation values in the polysomal fraction. Two adipogenesis relevant genes, FABP4 and WNT2, appeared to exhibit APA and differential 3′UTR length during differentiation in our previous study [12] by visually inspection. Here we confirmed these results by analytical methods. In our earlier work, the FABP4 gene exhibited a much longer 3′UTR under IN compared with CT conditions. The WNT2 gene in contrast showed the opposite behavior having a longer 3′UTR under CT conditions. Results obtained in this study showed a (positive) difference of 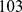 bases and a significant correlation value of 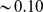 for the FABP4 gene, and for WNT2, a (negative) difference of 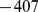 bases and a significance correlation value of 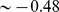.

A protein-protein network was previously described for pluripotent stem cells (Plurinet) [24]. Construction of the network was based on gene expression profiles for 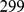 human proteins. We analyzed the distribution of differences in 3′UTR length for Plurinet genes having expression values in our dataset (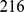 in 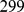). As shown in Fig. 4, the distribution of length differences substantially deviates from the behavior of all genes. In particular, genes with much longer 3′UTRs in control cells compared with induced cells were under represented. Additionally, we found an enrichment of the term “metabolism of non-coding RNA” among genes with 3′UTR length CT>IN, which could be associated with post-transcriptional regulation.

The dataset of Molina et al., was analyzed to understand the potential role for APA in protein changes [23]. Even though the cell line used by these authors was murine, this dataset was the most suitable available to compare with our RNAseq experiment. Several studies indicated a reasonable conservation in regulatory networks between human and mouse [29], [30]. Comparing differences between logFC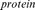 and the predicted protein quantity according to the logFC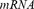 (logFC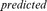), some large residuals (gene differences) were observed using this dataset (Fig. 1). Adipogenic relevant genes FABP4, GNS, TPM1, TPM3, KRT34, TMSB10 and ZYX were among genes with larger negative differences, i.e, logFC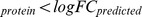. On the other hand, residuals with positive differences (logFC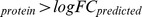), include LUM, PSAP, QSOX1, COL15A1, POSTN, ENPP2 and LPL (total RNA fraction). In addition, we have found that the observed differences (residuals) do not correlate significantly with the absolute magnitude of change in mRNA. As such the differences can't be explained by the expected compression of range (see section Appendix S1 (A)).

Clear differences were observed in APA isoform usage comparing IN and CT conditions, as well as differences between predicted fold change (by mRNA) and observed protein fold change for some genes. To further investigate this discrepancy we compared explained variances of base models just including logFC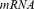 as predictive variable, against different models that incorporate miRNAs target site differences between transcripts as co-variables. The rationale behind including these miRNAs is to account for their potential effect on destabilizing or inhibiting translation resulting in discordance between the observed proteins and the mRNA levels. We have shown that hMSCs use their transcripts differentially during adipogenesis. We were able to test whether presence of miRNA binding sites is associated with change in the fate of specific transcripts by incorporating preferences for alternative transcripts (with alternative 3′UTR length) in our analyses. As summarized in Table 1, differences in explained variance were striking (even after adjusting for model complexity) when the effects of different miRNAs were introduced in the models. As expected, polysomal logFC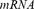 was higher correlated with logFC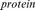 than the corresponding correlation in total RNA. This can be seen in the explained variances of both datasets, i.e., secreted and nuclear proteins. More surprising, however, is that changes in nuclear proteins were very poorly correlated with changes in mRNAs (the coefficient for logFC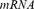 was never significant, even in absence of other co-variables). While several reasons might account for this, mechanisms involving protein translocation could be collaborating to this lack of correlation.

A range compression of logFC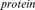 compared with logFC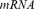 can be seen in the slope of Fig. 3 (A and C) and in coefficients for logFC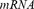 in Table 1. If translational efficiency decreases with increased mRNA levels (competition for scarce resources, e.g., ribosomes) in such a way that a linear trend is observed in log-log scale when plotting amounts of protein vs mRNA, the observed range compression would be expected (see section Appendix S1 (B)). In fact, this trend was observed in several studies [27], [27,32] and a coefficient of 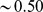 for *Saccharomyces cerevisiae* was determined [32]. We calculated a coefficient of 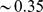 for comparisons with the secretome dataset, a reasonable estimate. We may be underestimating this coefficient since our comparisons and analyses are between species (mouse and human). Moreover, as we are only considering up to 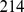 genes, our coefficient may not correspond to a global scenario in the cell. Finally, even though a significant improvement in explained variances is found by incorporating miRNAs in models, the small changes in logFC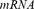 coefficients indicate that the improvement in performance is basically obtained by adjusting the prediction of “poorly-behaved” genes. In addition, the linear models presented here also reveal several genes whose regulation might be explained by specific miRNAs included in the models. In particular, we observed that the following genes were better fit by miRNA-models than the base model: ENPP2, LPL, FABP4, KRT14, TPM1, COL15A1 (polysomal RNA) and ENPP2, LPL, ADIPOQ, FABP5, FABP4, NID1, GSTM2, COL15A1, POSTN, KRT14 (total RNA). In the case of polysomal RNA, miR-130b and miR-558 were the miRNAs included in the model, whereas miR-150* was the co-variable in the model considering total RNA. It is worth mentioning, that we are only considering presence of miRNA binding sites, the expression levels of the miRNAs themselves is not included in our work.

Table 2 lists all significant miRNAs for which one-miRNA models were constructed, and also indicates which are previously mentioned as relevant for adipogenesis according to the revision of Zhang et al. [31]. In particular, we found 8 significant miRNAs of the 23 previously identified. Additionally, we found several miRNAs involved in other differentiation processes not described by Zhang et al. These include miR-142-3p, miR-16 and miR-15a which are associated with (TPA)-induced differentiation of human leukemia cells (HL-60) to monocyte/macrophage-like cells [37]. Also, miR-144 was implicated in erythroid differentiation [38] and miR-148a, miR-26, miR-378, miR-486 and miR-29 were identified in skeletal myogenic differentiation [39], and miR-10 was involved in endodermal differentiation [40]. Hence, miRNAs identified using our *in silico* analysis were previously found to be involved in several differentiation processes (including adipogenesis) by experimental methods.

Co-occurrence of miRNAs is not unusual; several miRNAs have been found to work together in gene regulation. Based on differences observed in alternative transcript usage, we explored miRNA co-occurrence in adipogenesis. We have found several strong associations in our presence/absence matrix weighted by differences in transcripts usage. Here we discuss some examples. Our primary analysis shows a statistically significant, but relatively trivial (since they are homologous) co-ocurrence of miR-204 and miR-211, whose common target is the Runx2 gene. miR-204/211 inhibits expression of Runx2, which inhibits osteogenesis and promotes adipogenesis of mesenchymal progenitor cells and bone marrow stromal cells [33]. We also observed a highly significant association of miRNA pair miR-17 and miR-93. They belong to the family including miR-17-5p, miR-20a, miR-93, and miR-106a, are differentially expressed in developing mouse embryos and have a controlling function in stem cell differentiation [41]. They are also key regulators of induced pluripotent stem cells and play a role in reprogramming efficiency of such cells [34]. On the other hand, miR-34 and miR-449 are negatively correlated in our data set implying that the presence of one results in the absence of the other. Both miRNAs belong to the same family; miR-449a, b and c are strong inducers of cell death, cell cycle arrest and cell differentiation; miR-34 is activated with expression of p53 protein and miR-449 is induced by E2F1, a cell cycle regulatory transcription factor. They are responsible for an asymmetric feedback loop that keeps the balance between E2F and p53 functions. miR-449 helps to ensure normal cell function but is also involved in maintaining a close interaction between cell differentiation and tumor suppression [35].

In summary, in the present work we found interesting and consistent differences in transcript isoforms used during adipogenesis. We found that, in general, induced cells had longer 3′UTRs compared with undifferentiated hMSCs. Furthermore, we characterized these differences by identifying genes whose transcripts had important differences in miRNAs target sites. Additionally, we demonstrated that by incorporating the effect of several miRNAs and alternative transcript usage in linear models, we were able to substantially improve prediction of logFC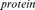 over the base model that only includes logFC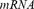. We need to expand our dataset by obtaining more accurate proteomic data to further corroborate our findings. Our results indicate that post-transcriptional regulation plays a key role in differentiation.

## Materials and Methods

### 1 Ethics statement

Samples were isolated and collected after obtention of written informed consent, agreeing with guidelines for research involving human subjects, and with the approval of the Ethics Committee of Fundação Oswaldo Cruz, Brazil (approval number 419/07), as previously mentioned in [12].

### 2 Sample description

We used samples described by Spangenberg *et al.* [12]. Raw data is available under the accession number E-MTAB-1366 in the ArrayExpress repository. Stem cells were obtained from adipose tissue of three obese human donors. hASCs were isolated, cultured and characterized as previously described [42]. Briefly, adipogenesis was induced with 6 day-cycles of induction/maintenance over 21 days. Induction medium contained the adipogenic inducers insulin, dexamethasone, indomethacin and IBMX; maintenance medium contained insulin. Medium was changed every 3 days. The degree of adipogenic differentiation was determined by assessing cytoplasmic accumulation of triglycerides by staining with Oil Red O or Nile Red (Sigma-Aldrich). Samples were taken at time point 0 (control samples, CT) and then after three days (induced samples, IN).

A total of 13 samples were sequenced with SOLiD4 System (Applied Biosystems), 7 CT (2 polysomal-associated RNA and 6 total RNA samples) and 6 IN (3 polysomal-associated RNA and 3 total RNA). Table 3 shows an overview of samples. The proteomic data used in this study is from Molina *et al.* [23]. They quantified two sets of 3T3-L1 murine proteins with SILAC: 280 nuclear and 147 secreted proteins, with a total of 427 proteins. These were analyzed during adipogenesis (at day 0, 1, 3, 5 and 7).

**Table 3:** Mapping statistics of RNA-seq.

donor	condition	raw data	reads for mapping	mapped	unmapped	junctions	%
61	CT_poly	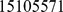	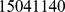	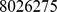	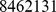	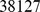	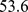
61	IN_poly	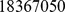	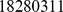	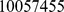	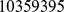	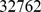	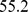
67	CT_poly	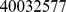	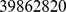	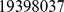	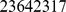	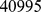	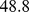
67	IN_poly	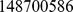	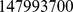	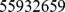	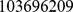	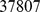	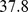
67	CT_total	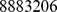	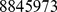	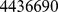	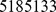	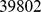	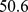
70	CT_poly	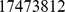	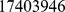	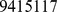	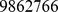	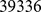	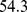
70	IN_poly	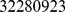	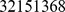	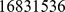	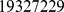	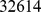	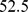
70	CT_total	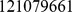	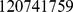	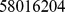	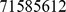	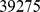	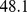
61	IN_total	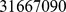	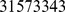	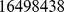	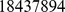	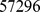	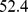
67	IN_total	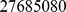	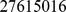	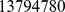	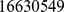	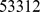	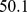
70	IN_total	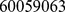	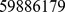	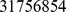	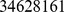	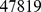	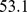
61	CT_total	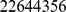	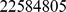	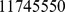	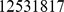	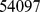	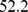
67	CT_total	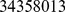	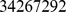	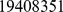	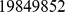	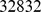	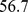

Mapping data of SOLiD runs. Following data is shown: donor number, condition considered (CT or IN, and polysomal or total RNA), number of raw reads obtained from the sequencing process, number of reads considered for mapping, number of mapped reads, unmapped reads, and the percentage of mapped reads.

While our RNA-seq data is from human donors, nevertheless we decided to compare it against murine proteomic data. Of course, this assumes a high conservation at protein level between this two organisms in the involved networks, a fact relatively supported by recent studies [29], [30]. Furthermore, at transcriptional level, some studies have shown that a conservation is also seen for several genes [43].

### 3 Primary analysis of SOLiD RNA-seq samples

Table 3 summarizes results of the mapping procedure with *tophat2* and *cuffdiff*. We obtained a median of 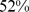 mapped reads in the 13 samples. Information on transcript usage for 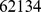 ensembl gene ids was obtained from *cuffdiff* for total and polysomal RNA samples. These were filtered according to the quality status of transcripts, because the low number of reads might compromise determination of FPKM. After filtering we obtained 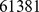 for both sets, polysomal and total RNA. From those genes, 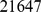 have annotated 3′UTRs according to ensembl annotation, corresponding to 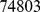 transcripts.

### 4 Summarizing transcript differences

We calculated the relative frequency of each transcript for each condition (IN and CT), and weighted the transcript 3′UTR length by the differences in frequency (we did this for each gene). To assess the significance of the differences observed above, we tested our data using the Cochran-Mantel-Haenszel statistic, a test of linear trend alternative to independence [26], which is more sensitive than a standard 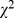 test if a linear trend holds. Additionally, for each gene we calculated and analyzed the Pearson-r distribution between 3′UTR length and condition (CT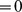, IN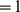) [26].

### 5 Mapping and annotation


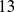
 samples were mapped onto the reference genome (hg19 GR37p2) using *tophat2* [44]. *cufflinks* [45] v2.1.1 was then used for transcript assembly. Determination of isoform abundance was done with *cuffdiff* v2.1.1. The annotation file used for counting was based on the genome version Hg19 Gr37p10 (August 2012), downloaded from the ensembl. The 3′UTR annotation file was also created from the ensembl (version Hg19Gr37p10, 15 August 2012) human gff annotation file. The miRNA target information considered is the one included in the R package microRNA, from Gentleman and Falcon [46], which is also based on ensembl. Currently, it contains a total of 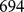 miRNAs targeting a total of 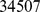 transcripts.

Mapping, gene expression assessment and differential expression determination in our earlier work was performed using the *Rsubread* and *edgeR* R packages.

### 6 Linear model for correlation of microRNAs with protein levels

We developed a linear model approach to show the influence of miRNAs targeting 3′UTR regions of transcripts on respective protein expression levels.

**Figure 6:**
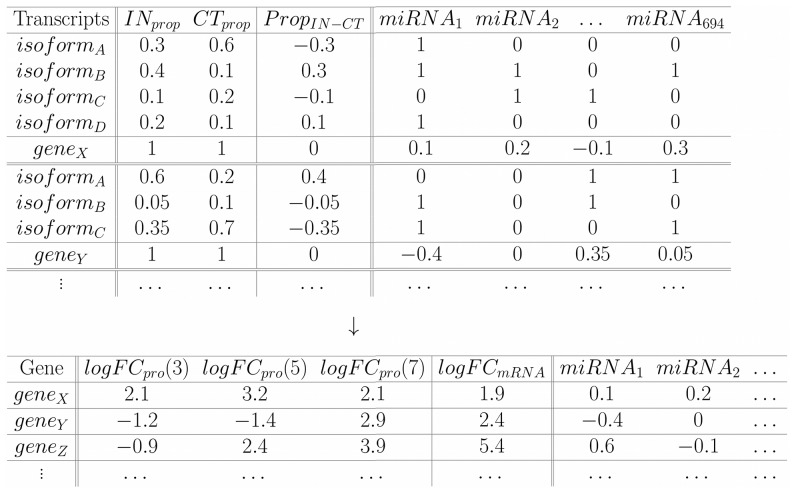
Representative table for constructing the model. For each gene we determined the proportion of FPKM in each sample and calculated the differences (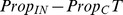). Furthermore, we determined the miRNAs targeting transcripts (inside 3′UTRs). A total of 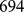 were considered. The isoform has a 1 in 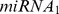 if that miRNA is present in that transcript, a 0 otherwise. For each 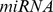 (eg. 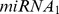) corresponding to one gene (e.g. 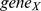), the 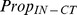 vector is multiplied by the presence/absence vector of 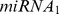 (with assigned 1 s and 0 s). The intermediate result is, thus, a vector having the respective 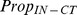 value if 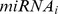 was present in the isoform and 0 otherwise (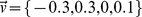). The resulting vector 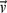 is summed giving a total value for 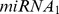 for 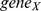 (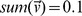). This represents the mean weighted usage of the miRNA in that specific gene. Larger positive values indicate that the miRNA is used more (appears more often) in IN than in CT. Larger negative values represent a higher usage in CT (values around 0 indicate same usage in both). The same procedure is done for each miRNA (so a vector of 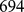 values is assigned to 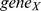) and for each gene. The gene wise table below in addition to showing the resulting values calculated above, also shows the other data needed for the model; the logFC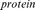 values (at day 3, 5 and 7, from Molina *et al.*) and the respective logFC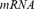 values (our data).

Our starting point is data generated from *cuffdiff* software. An abundance normalized measure, FPKM, is first obtained for each transcript isoform which represents the number of fragments per kilobase per million fragments falling on each feature (e.g., transcript). A FPKM value is calculated for each condition and each transcript, which allows determination of differential isoform usage. The proportion of each transcript isoform for each gene was determined under all conditions based on the FPKM values. Proportions in control samples are subtracted from the proportions in induced samples (IN) to determine the differences in isoform usage. Differences in proportions of each isoform for each gene (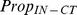) and the presence of miRNA binding sites in transcript 3′UTRs (represented as 1 s in Fig. 6) were determined. The 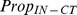 value is multiplied by the corresponding miRNA binding site present and the resulting vector is summed for a given gene (Fig. 6). This results in one value for each miRNA binding site for each gene, which represents a weighted mean for usage of that miRNA for that gene. Large positive values (closer to 1) are miRNAs highly used in IN samples, large negative values (closer to −1) are those most used in CT. In other words, values closer to 1 correspond to miRNAs targeting transcripts preferentially used in IN samples, and those with values closer to −1 are preferentially used in CT. Note that a given miRNA might have several binding sites in a given 3′UTR, nevertheless we considered one or more sites as either present or absent with no multiplicity value assigned. This is still a matter open for discussion, since several studies have shown cooperative effects in the past [47]–[50], while others suggested the opposite behavior in large and comprehensive human and mouse datasets [18], [51]. We have also run our analysis considering the cooperative effect, obtaining conceptually similar results (data not shown). However, for simplicity reasons, we decided to consider the simplest model accepted and used the present/absent values. Since such values are determined for each gene and for each miRNA, results can be presented in a table with 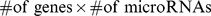. For each day d (1, 3, 5 and 7), miRNA *i* and assuming 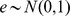, we applied following model:
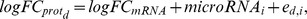
so we can determine the effect of each microRNA on protein level.

The possibility that significant miRNAs coefficients arise by chance was assessed by bootstrap analysis. We randomly assigned the existing values to genes for each miRNA, and calculated the explained variance from the linear model. We repeated this procedure 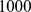 times. The proportion of times the variance explained by the random model was larger than the “true” model was determined for each miRNA for the four datasets (nuclear, secreted vs total, polysomal). We arbitrarily set a threshold of 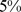 (times the random wins over the “true”) for each dataset and compared the explained variances of the two groups (random vs. “true”) using the Kruskal-Wallis test.

### 7 Determining significative correlation for co-occurring microRNAs

Co-occurrence of miRNAs was investigated to demonstrate regulatory effects. We analyzed the complete presence/absence table of miRNAs in human (downloaded from the *microRNA* R package). This table contains all transcripts analyzed (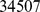) in which 1 is assigned if microRNA*_i_* is present in that transcript, and a 0 if not, for all miRNAs considered (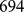). We compared pairwise correlations for all miRNAs based on that information and the same in our weighted data set. This means, we also determined the correlation of miRNAs, but weighted by proportion of the transcripts used. If a transcript with a given miRNA is used only 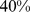 of the time by the gene, the miRNA value assigned would be 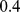, and not a simple 1.

Not all entries were used for each pairwise correlation; we eliminate all entries in which both miRNAs had values of 0, i.e., pairwise-zero entries. Several of such entries exists, since not every transcript has either one of the miRNAs considered (in most cases, they have neither). With such strategy we have compared the correlations found by the presence/absence table, and the ones obtained by our weighted filtered data.

## Supporting Information

Figure S1
**Heatmap of the residuals of the model logFC** 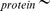 **logFC** 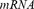 **of nuclear proteins.** Protein levels (logFC) of the set of nuclear proteins are compared against the logFC of our data set and the residuals of the linear model analyzed; polysomal fraction (A) and total fraction (B). All time points are considered: day 1, 3, 5 and 7 (dendrogram on the top). Genes are on the rows (dendrogram on the left). Only data for genes with large absolute residuals are shown.(TIFF)

Figure S2
**Box plot to show the distribution of random and “true” models in the bootstrap.** All comparisons are shown (polysomal-secreted, polysomal-nuclear, total-secreted, total-nuclear). For each such dataset, bootstrap was performed, and two groups were determined. Low-Random group holds models in which “true” miRNAs data won over random sampling of the miRNA values at least 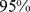 of the time. The High-Random group corresponds to miRNAs in which random sampling of miRNA values produce models that are better than the “true” more than 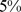 of the time.(TIFF)

Appendix S1(A) Range compression is observed in protein log fold-change (in our data), when logFC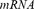 is considered as predictor. The size of this effect is the translational efficiency (in log-log scale) as a function of the quantity of mRNA. (B) Messenger exponential decay with alternative target miRNA sites. We show that the basic assumption underlying the way in which we modeled the effect of miRNAs is an exponential decay of mRNA as a function of differential target sites.(PDF)
