# Correction: Role of Alternative Polyadenylation during Adipogenic Differentiation: An *In Silico* Approach

**DOI:** 10.1371/journal.pone.0091409

**Published:** 2014-05-30

**Authors:** The PLOS ONE Staff

The legends for Figure 2 and Figure 3 are incorrectly switched. Please see the complete, corrected Figure 2 here.

**Figure 2:**
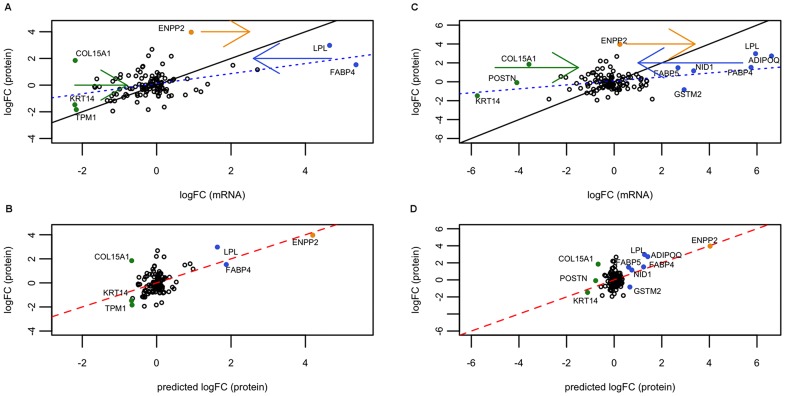
Linear models for day 5 secreted proteins represented graphically. (A, B) Polysomal fraction, (C, D) total RNA. (A) and (C): plot representing logFC_mRNA_ against logFC_protein_. The dashed blue line is the best fitting line of the base model, logFC_protein_ against logFC_mRNA_. The straight black line is the identity line (so you get an idea of the real coefficient of the model). The colored full dots are genes, which are moved after applying the model with miRNAs. Hence, they represent genes that are better explained by our model. The arrows indicate the direction of the movement. (B) and (D): plot representing our linear model including miRNA effect. In this case, the best (multivariate) model is shown: miR-130b and miR-558 (polysomal) and miR-150* (total). Full dots are the genes that were corrected by our model, being now closer to the protein prediction line of the model (red full line). Black identity line concurs with the red line. Note that the abscissas of (A) and (C) seem to have a compression of range with respect to the plots below, (B) and (D). This is not a compression, since they are different x-axis: (A) and (C) hold logFC_mRNA_ values, while (B) and (D) logFC_protein_.

Please see the complete, corrected Figure 3 here.

**Figure 3:**
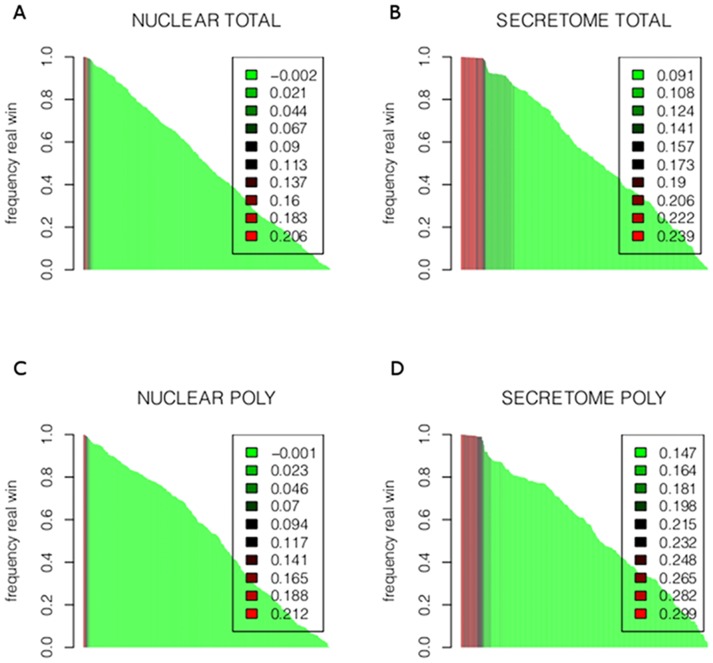
Bootstrap to asses our results for each RNA fraction and each protein set. Bootstrap results for total RNA fractions are shown in A (nuclear) and B (secretome). Polysomal fraction is shown in C (nuclear) and D (secretome). For each such pair of conditions, we performed a bootstrap analysis as explained in 6. For each miRNA we permute the values of the genes and calculate the explained variance from the resulting linear model. This procedure is repeated 1000 times. The y-axis represents how many times the “true” miRNA wins over the random model. The x-axis represents all miRNAs. The colors, from red to green, represent the explained variance from the current “true” model. It can be observed that the miRNAs win almost all times (the larger bars, almost reaching 1), explain the larger variance, and hence produce the best models (red).
