# Supplementary figures and images for: Role of Alternative Polyadenylation during Adipogenic Differentiation: An In Silico Approach
Source: PLoS One. 2013 Oct 15;8(10):e75578. doi: 10.1371/journal.pone.0075578 (PMC3797115; doi:10.1371/journal.pone.0075578)

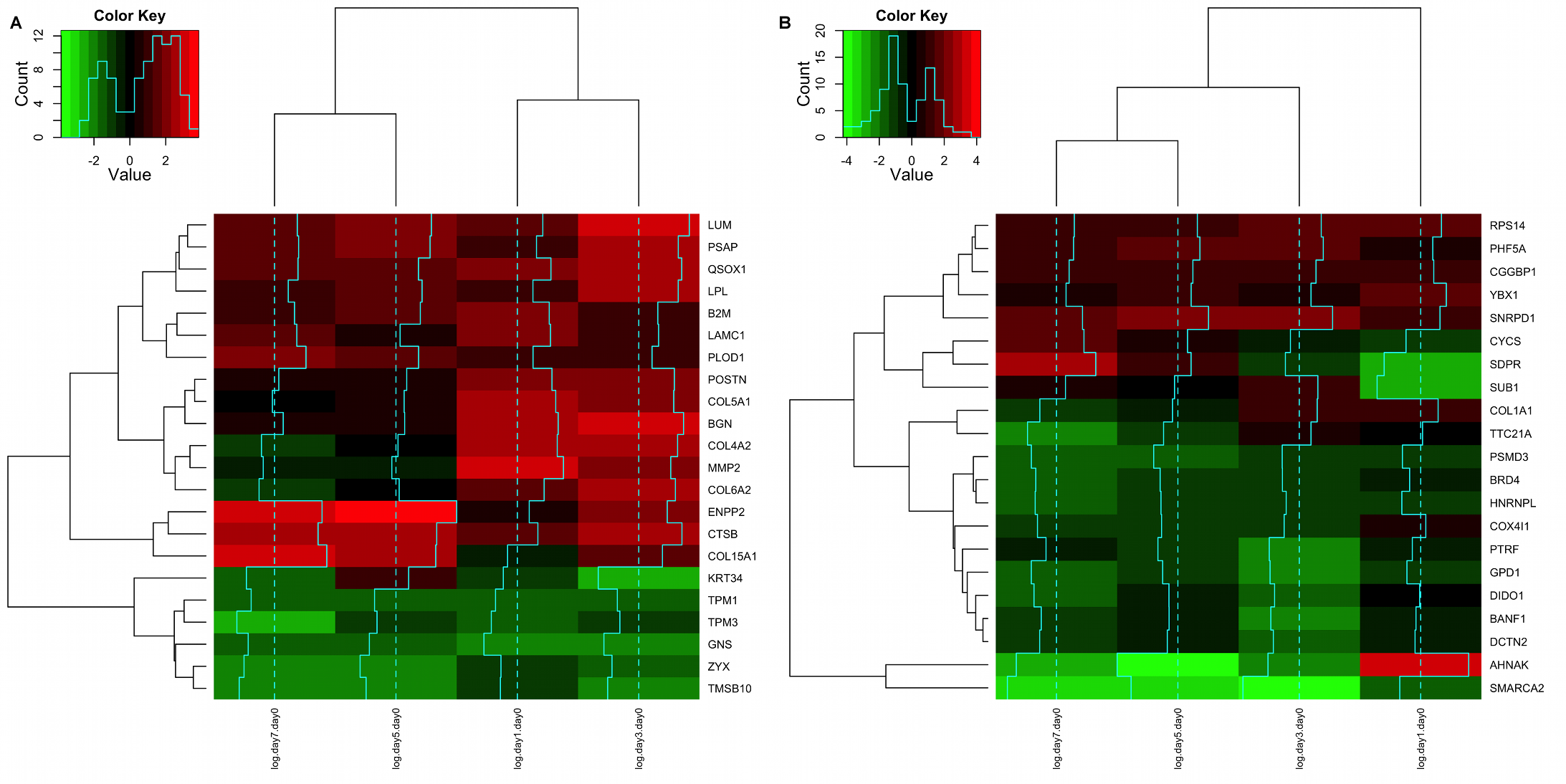

Supplement: Figure S1 — Heatmap of the residuals of the model logFC logFC of nuclear proteins. Protein levels (logFC) of the set of nuclear proteins are compared against the logFC of our data set and the residuals of the linear model analyzed; polysomal fraction (A) and total fraction (B). All time points are considered: day 1, 3, 5 and 7 (dendrogram on the top). Genes are on the rows (dendrogram on the left). Only data for genes with large absolute residuals are shown. (TIFF) [file pone.0075578.s001.tif]

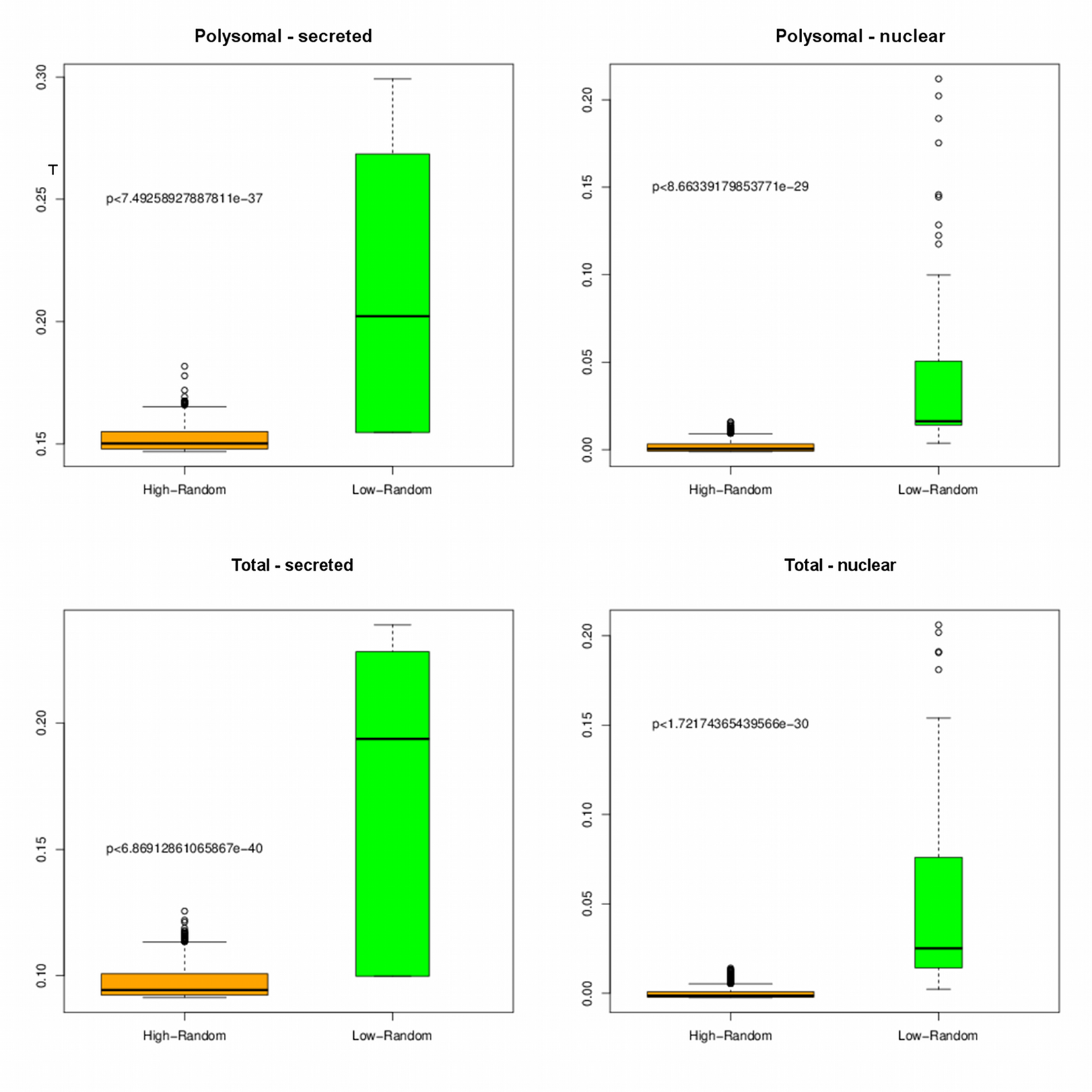

Supplement: Figure S2 — Box plot to show the distribution of random and “true” models in the bootstrap. All comparisons are shown (polysomal-secreted, polysomal-nuclear, total-secreted, total-nuclear). For each such dataset, bootstrap was performed, and two groups were determined. Low-Random group holds models in which “true” miRNAs data won over random sampling of the miRNA values at least of the time. The High-Random group corresponds to miRNAs in which random sampling of miRNA values produce models that are better than the “true” more than of the time. (TIFF) [file pone.0075578.s002.tif]
