# Supplementary material for: Role of Alternative Polyadenylation during Adipogenic Differentiation: An In Silico Approach
Source: PLoS One. 2013 Oct 15;8(10):e75578. doi: 10.1371/journal.pone.0075578 (PMC3797115; doi:10.1371/journal.pone.0075578)
Supplement: Appendix S1 — (A) Range compression is observed in protein log fold-change (in our data), when logFC is considered as predictor. The size of this effect is the translational efficiency (in log-log scale) as a function of the quantity of mRNA. (B) Messenger exponential decay with alternative target miRNA sites. We show that the basic assumption underlying the way in which we modeled the effect of miRNAs is an exponential decay of mRNA as a function of differential target sites. (PDF) [file pone.0075578.s003.pdf]

Appendix

A Range compression effect

Studies in human and yeast found a linear trend for logarithm protein abundance versus logarithmic mRNA abundance [1–3]. A reasonable fit for a linear model between both (log-transformed) variables is obtained in which protein abundance can be described as:

$$\ln(protein) = b_1 \cdot \ln(mRNA) + b_0,$$

where the coefficient b_1 represents the translational efficiency as function of the quantity of mRNA, and b_0 is the intercept that can accommodate all other additive effects in logarithmic scale (multiplicative in the original scale), as presented by Stevens and Brown [3] or Tuller *et al.* [2]. Following this, the best predictor of the quantity of protein is:

$$protein = e^{b_1 \cdot \ln(mRNA) + b_0}$$

In differential expression experiments we usually compare two conditions, x and y. Hence:

$$protein_x = e^{b_1 \cdot \ln(mRNA_x) + b_0}$$

and

$$protein_y = e^{b_1 \cdot \ln(mRNA_y) + b_0}$$

Comparisons in this field are usually made through a $log-$ratio to obtain:

$$\frac{protein_x}{protein_y} = \frac{e^{b_1 \cdot \ln(mRNA_x) + b_0}}{e^{b_1 \cdot \ln(mRNA_y) + b_0}}$$

and after taking the log values on both sides:

$$\ln \frac{protein_x}{protein_x} = \ln \frac{e^{b_1 \cdot \ln(mRNA_x) + b_0}}{e^{b_1 \cdot \ln(mRNA_y) + b_0}} = \ln(e^{b_1 \cdot \ln(mRNA_x) + b_0}) - \ln(e^{b_1 \cdot \ln(mRNA_y) + b_0})$$
$$= (b_1 \cdot \ln(mRNA_x) + b_0) - (b_1 \cdot \ln(mRNA_y) + b_0) = b_1 \cdot (\ln(mRNA_x) - \ln(mRNA_y))$$

$$\Leftrightarrow \ln \frac{protein_x}{protein_y} = b_1 \cdot \ln \frac{mRNA_x}{mRNA_y}$$

This shows a range compression of size b_1 in protein log fold-change (the data we are considering), when logFC_{mRNA} is considered as predictor. Furthermore, the size of this effect (coefficient b_1) is the translational efficiency (in log-log scale) as a function of the quantity of mRNA. Additionally, as b_1 was derived from a log-log regression it is scale invariant, with effect of the scale represented by b_0 that is removed in log fold-change comparisons.

B Messenger exponential decay with alternative target miRNA sites

While model comparisons is beyond the scope of this study, we show that the basic assumption underlying the way in which we modeled the effect of miRNAs is an exponential decay of mRNA as function of differential target sites. If we assume that the linear log-log relationship between protein and mRNA holds, we can introduce the effect of a miRNA as:

$$\ln(protein) = b_0 + b_1 \cdot \ln(mRNA) + b_2 \cdot miR,$$

where miR is the proportion of reads assigned to a given mRNAs (for a given gene) that have a recognition site for this miRNA. As shown previously, now the best predictor of the quantity of protein is:

$$protein = e^{b_0+b_1\cdot\ln(mRNA)+b_2\cdot miR}$$

When we compare two conditions, x and y as in the previous subsection, we have:

$$\frac{protein_x}{protein_y} = \frac{e^{b_0+b_1\cdot\ln(mRNA_x)+b_2\cdot miR_x}}{e^{b_0+b_1\cdot\ln(mRNA_y)+b_2\cdot miR_y}} = \frac{e^{b_1\cdot\ln(mRNA_x)}}{e^{b_1\cdot\ln(mRNA_y)}} \cdot \frac{e^{b_2\cdot miR_x}}{e^{b_2\cdot miR_y}}$$

.

Rearranging the terms, we can write this relation as:

$$\frac{protein_x}{protein_y} = \left(\frac{mRNA_x}{mRNA_y}\right)^{b_1} \cdot e^{b_2\cdot(miR_x - miR_y)}$$

which is an exponential function of the differences in alternative target sites between the two conditions x and y. In the common form of log fold-changes, we have:

$$\ln\frac{protein_x}{protein_y} = b_1 \cdot \ln\frac{mRNA_x}{mRNA_y} + b_2 \cdot (miR_x - miR_y),$$

that is, the model we have used in our setting.

References

1. Futch B, Latter GI, Monardo P, McLaughlin CS, Garrels JI (1999) A sampling of the yeast proteome. Molecular and celular biology 19: 7357–7368.

2. Tuller T, Kupiec M, Ruppin E (2007) Determinants of protein abundance and translation efficiency in S. *cerevisiae*. PLoS Computational Biology 3: 10.

3. Stevens SG, Brown CM (2013) *In silico* estimation of translation efficiency in human cell lines: potential evidence for widespread translational control. PLoS One 8: e57625.
